# Supplementary material for: Detection of Pathways Affected by Positive Selection in Primate Lineages Ancestral to Humans
Source: Mol Biol Evol. 2017 Feb 25;34(6):1391–402. doi: 10.1093/molbev/msx083 (PMC5435107; doi:10.1093/molbev/msx083)
Supplement: Supplementary Data [file msx083_Supp.zip › Figure S6.pdf]

Figure S6

Heat maps showing branch specific ΔlnL4 scores of genes in pathways that score significant ($q < 0.2$) in the gene set enrichment test after pruning. The ΔlnL4 score is computed as the fourth root of the log-likelihood ratio in the branch-site test for positive selection. Branches where a pathway scores significant are marked with a '*'. The genes are grouped by hierarchical clustering to visualize blocks with similar signals among branches. Genes for which ΔlnL4 scores were not available (NA) in a certain branch are depicted in grey. Genes are merged (horizontally) with their paralog(s) into an 'ancestral gene' in the branches preceding a duplication and their scores were included only once in the calculation of the SUMSTAT score for these branches. Genes with (vertically) merged branches represent cases where the sequence of one or more species is missing or excluded, resulting in a single 'average' ΔlnL4 score over multiple branches. We used this score when testing each branch separately. Full-size versions of these heat maps are available at https://dx.doi.org/10.6084/m9.figshare.3119026.

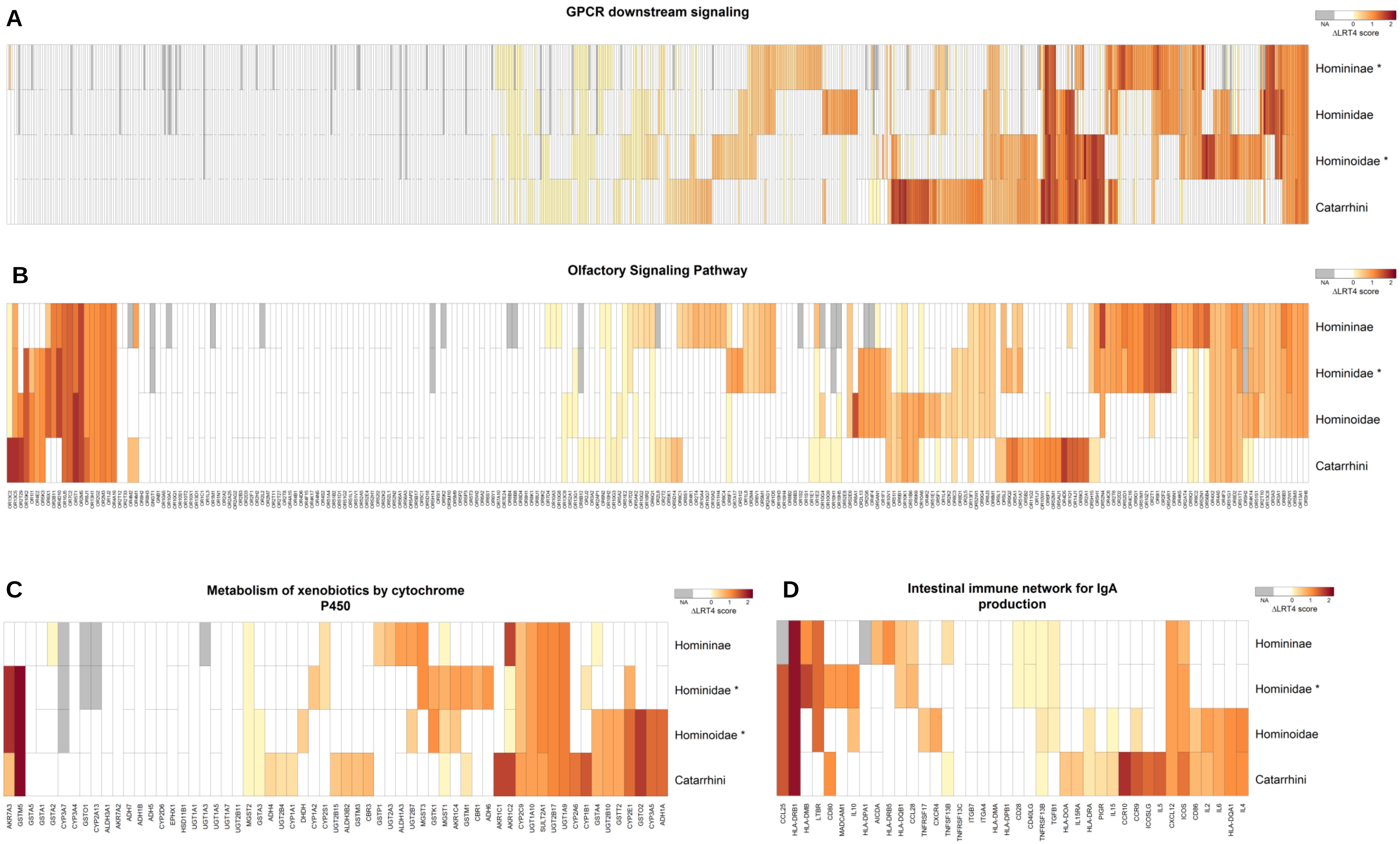

A
GPCR downstream signaling
NA 0 1 2
ΔLRT4 score
Homininae *
Hominidae
Hominoidae *
Catarrhini
B
Olfactory Signaling Pathway
NA 0 1 2
ΔLRT4 score
Homininae
Hominidae *
Hominoidae
Catarrhini
C
Metabolism of xenobiotics by cytochrome P450
NA 0 1 2
ΔLRT4 score
Homininae
Hominidae *
Hominoidae *
Catarrhini
AKR7A3 GSTM5 GSTA5 GSTA1 GSTA2 CYP3A7 CYP3A4 GSTO1 CYP2A13 ALDH3A1 AKR7A2 ADH7 ADH1B ADH5 CYP2D6 EPHX1 HSD11B1 UGT1A1 UGT1A3 UGT1A5 UGT1A7 UGT2B11 MGST2 GSTA3 ADH4 UGT2B4 CYP1A1 DHDH CYP1A2 CYP2S1 UGT2B15 ALDH3B2 GSTM3 CBR3 GSTP1 UGT2A3 ALDH1A3 UGT2B7 MGST3 GSTK1 MGST1 AKR1C4 GSTM1 CBR1 ADH6 AKR1C1 AKR1C2 CYP2C9 UGT1A10 SULT2A1 UGT2B17 UGT1A9 CYP2A6 CYP1B1 GSTA4 UGT2B10 GSTT2 CYP2E1 GSTO2 CYP3A5 ADH1A
D
Intestinal immune network for IgA production
NA 0 1 2
ΔLRT4 score
Homininae
Hominidae *
Hominoidae
Catarrhini
CCL25 HLA-DRB1 HLA-DMB LTBR CD80 MADCAM1 IL10 HLA-DPA1 AICDA HLA-DRB5 HLA-DQB1 CCL28 TNFRSF17 CXCR4 TNFSF13B TNFRSF13C ITGB7 ITGA4 HLA-DMA HLA-DPB1 CD28 CD40LG TNFRSF13B TGFB1 HLA-DOA IL15RA HLA-DRA PIGR IL15 CCR10 CCR9 ICOSLG IL5 CXCL12 ICOS CD86 IL2 IL6 HLA-DQA1 IL4

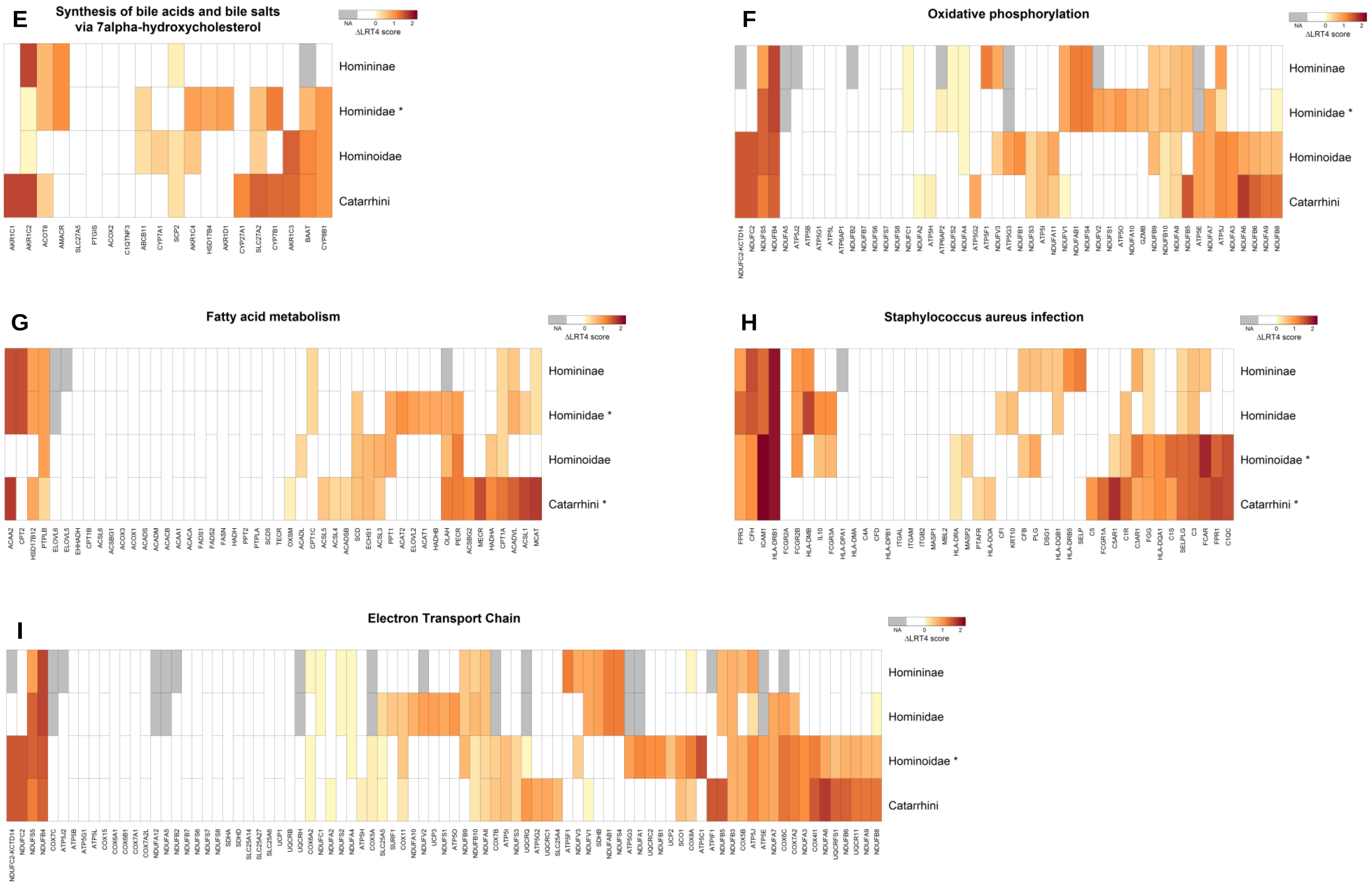
E
Synthesis of bile acids and bile salts via 7alpha-hydroxycholesterol
NA 0 1 2
ΔLRT4 score
Homininae
Hominidae *
Hominoidae
Catarrhini
F
Oxidative phosphorylation
NA 0 1 2
ΔLRT4 score
Homininae
Hominidae *
Hominoidae
Catarrhini
G
Fatty acid metabolism
NA 0 1 2
ΔLRT4 score
Homininae
Hominidae *
Hominoidae
Catarrhini *
H
Staphylococcus aureus infection
NA 0 1 2
ΔLRT4 score
Homininae
Hominidae
Hominoidae *
Catarrhini *
I
Electron Transport Chain
NA 0 1 2
ΔLRT4 score
Homininae
Hominidae
Hominoidae *
Catarrhini

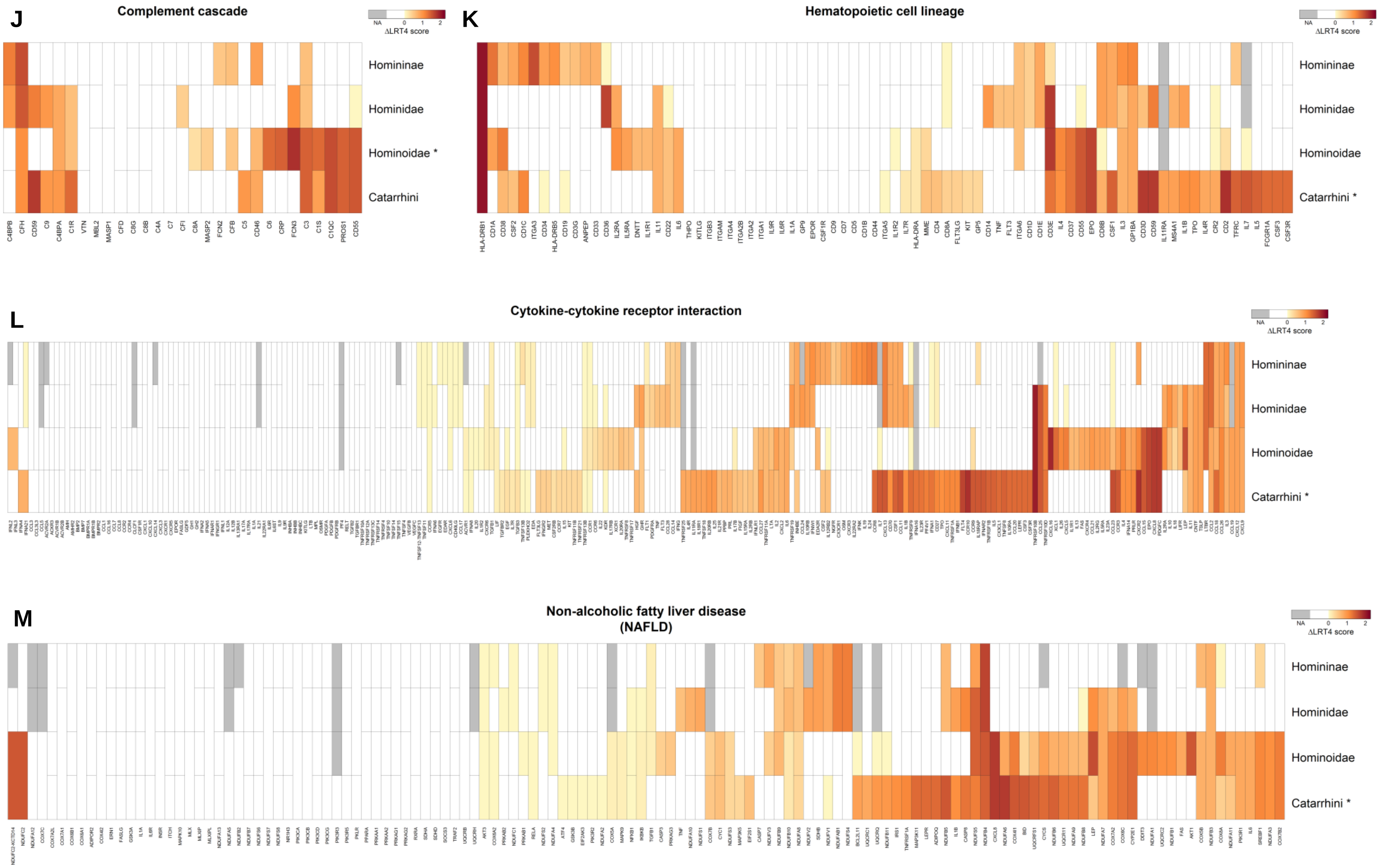

J
Complement cascade
NA 0 1 2
ΔLRT4 score
Homininae
Hominidae
Hominoidae *
Catarrhini
C4BPB CFH CD59 C9 C4BPA C1R VTN MBL2 MASP1 CFD C8G C8B C4A C7 CFI C8A MASP2 FCN2 CFB C5 CD46 C6 CRP FCN3 C3 C1S C1QC PROS1 CD55
K
Hematopoietic cell lineage
NA 0 1 2
ΔLRT4 score
Homininae
Hominidae
Hominoidae
Catarrhini *
HLA-DRB1 CD1A CD38 CSF2 CD1C ITGA3 CD34 HLA-DRB5 CD19 CD3G ANPEP CD33 CD36 IL2RA IL5RA DNTT IL1R1 IL11 CD22 IL6 THPO KITLG ITGB3 ITGAM ITGA4 ITGA2B ITGA2 ITGA1 IL9R IL6R IL1A GP9 EPOR CSF1R CD9 CD7 CD5 CD1B CD44 ITGA5 IL1R2 IL7R HLA-DRA MME CD4 CD8A FLT3LG KIT GP5 CD14 TNF FLT3 ITGA6 CD1D CD1E CD3E IL4 CD37 CD55 EPO CD8B CSF1 IL3 GP1BA CD3D CD59 IL11RA MS4A1 IL1B TPO IL4R CR2 CD2 TFRC IL7 IL5 FCGR1A CSF3 CSF3R
L
Cytokine-cytokine receptor interaction
NA 0 1 2
ΔLRT4 score
Homininae
Hominidae
Hominoidae
Catarrhini *
M
Non-alcoholic fatty liver disease
(NAFLD)
NA 0 1 2
ΔLRT4 score
Homininae
Hominidae
Hominoidae
Catarrhini *

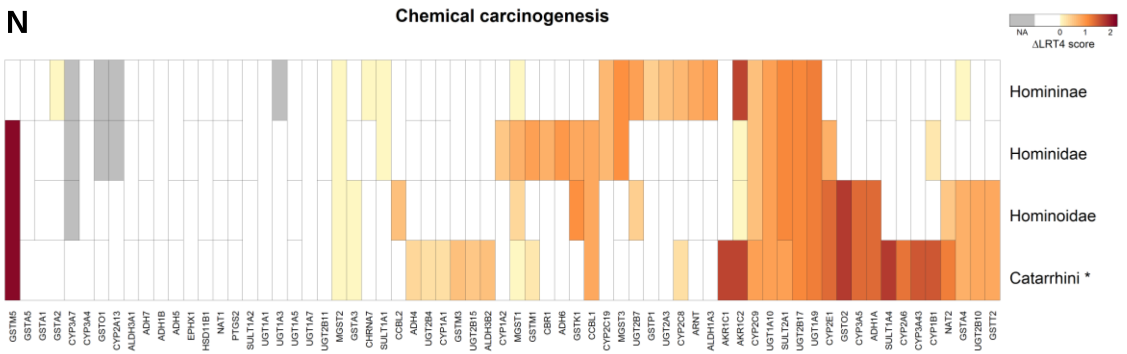
N
Chemical carcinogenesis
NA 0 1 2
ΔLRT4 score
Homininae
Hominidae
Hominoidae
Catarrhini *
GSTM5 GSTA5 GSTA1 GSTA2 CYP3A7 CYP3A4 GSTO1 CYP2A13 ALDH3A1 ADH7 ADH1B ADH5 EPHX1 HSD11B1 NAT1 PTGS2 SULT1A2 UGT1A1 UGT1A3 UGT1A5 UGT1A7 UGT2B11 MGST2 GSTA3 CHRNA7 SULT1A1 CCBL2 ADH4 UGT2B4 CYP1A1 GSTM3 UGT2B15 ALDH3B2 CYP1A2 MGST1 GSTM1 CBR1 ADH6 GSTK1 CCBL1 CYP2C19 MGST3 UGT2B7 GSTP1 UGT2A3 CYP2C8 ARNT ALDH1A3 AKR1C1 AKR1C2 CYP2C9 UGT1A10 SULT2A1 UGT2B17 UGT1A9 CYP2E1 GSTO2 CYP3A5 ADH1A SULT1A4 CYP2A6 CYP3A43 CYP1B1 NAT2 GSTA4 UGT2B10 GSTT2

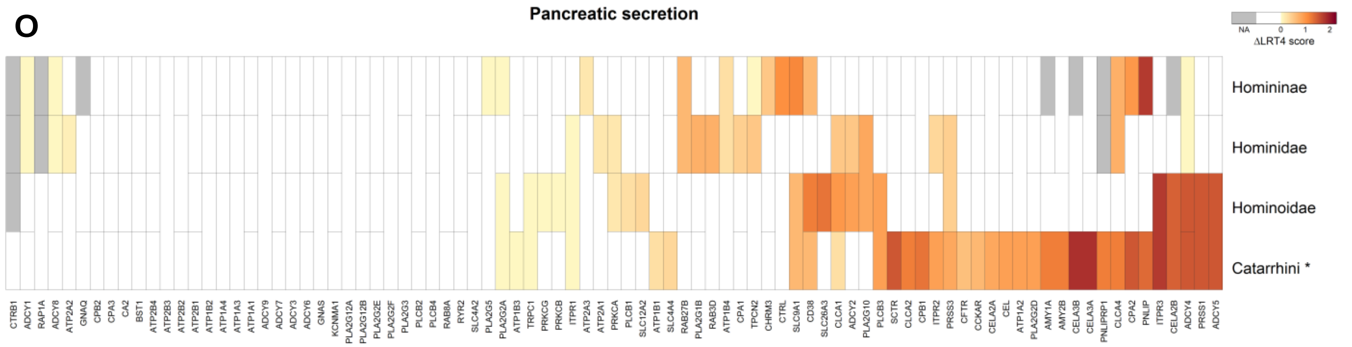
O
Pancreatic secretion
NA 0 1 2
ΔLRT4 score
Homininae
Hominidae
Hominoidae
Catarrhini *
CTRB1 ADCY1 RAP1A ADCY8 ATP2A2 GNAQ CPB2 CPA3 CA2 BST1 ATP2B4 ATP2B3 ATP2B2 ATP2B1 ATP1B2 ATP1A4 ATP1A3 ATP1A1 ADCY9 ADCY7 ADCY3 ADCY6 GNAS KCNMA1 PLA2G12A PLA2G12B PLA2G2E PLA2G2F PLA2G3 PLCB2 PLCB4 RAB8A RYR2 SLC4A2 PLA2G5 PLA2G2A ATP1B3 TRPC1 PRKCG PRKCB ITPR1 ATP2A3 ATP2A1 PRKCA PLCB1 SLC12A2 ATP1B1 SLC4A4 RAB27B PLA2G1B RAB3D ATP1B4 CPA1 TPCN2 CHRM3 CTRL SLC9A1 CD38 SLC26A3 CLCA1 ADCY2 PLA2G10 PLCB3 SCTR CLCA2 CPB1 ITPR2 PRSS3 CFTR CCKAR CELA2A CEL ATP1A2 PLA2G2D AMY1A AMY2B CELA3B CELA3A PNLIPRP1 CLCA4 CPA2 PNLIP ITPR3 CELA2B ADCY4 PRSS1 ADCY5

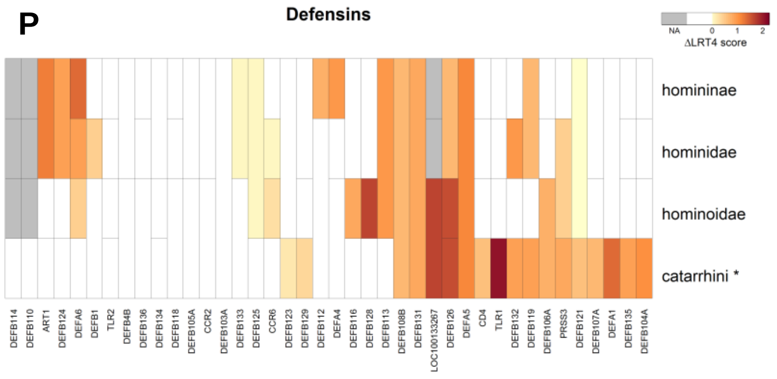
P
Defensins
NA 0 1 2
ΔLRT4 score
homininae
hominidae
hominoidae
catarrhini *
DEFB114 DEFB110 ART1 DEFB124 DEFA6 DEFB1 TLR2 DEFB4B DEFB136 DEFB134 DEFB118 DEFB105A CCR2 DEFB103A DEFB133 DEFB125 CCR6 DEFB123 DEFB129 DEFB112 DEFA4 DEFB116 DEFB128 DEFB113 DEFB108B DEFB131 LOC100133267 DEFB126 DEFA5 CD4 TLR1 DEFB132 DEFB119 DEFB106A PRSS3 DEFB121 DEFB107A DEFA1 DEFB135 DEFB104A

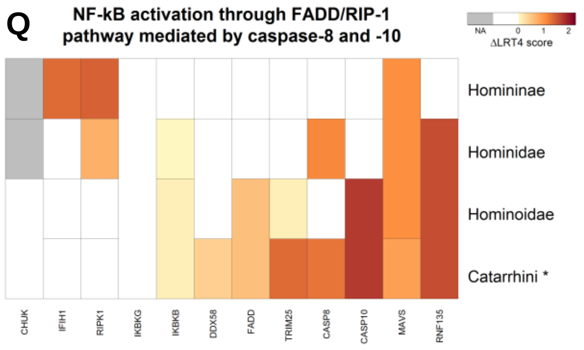
Q
NF-kB activation through FADD/RIP-1
pathway mediated by caspase-8 and -10
NA 0 1 2
ΔLRT4 score
Homininae
Hominidae
Hominoidae
Catarrhini *
CHUK IFIH1 RIPK1 IKBKG IKBKB DDX58 FADD TRIM25 CASP8 CASP10 MAVS RNF135
