# Supplementary material for: Detection of Pathways Affected by Positive Selection in Primate Lineages Ancestral to Humans
Source: Mol Biol Evol. 2017 Feb 25;34(6):1391–402. doi: 10.1093/molbev/msx083 (PMC5435107; doi:10.1093/molbev/msx083)
Supplement: Supplementary Data [file msx083_Supp.zip › Figure S8.pdf]

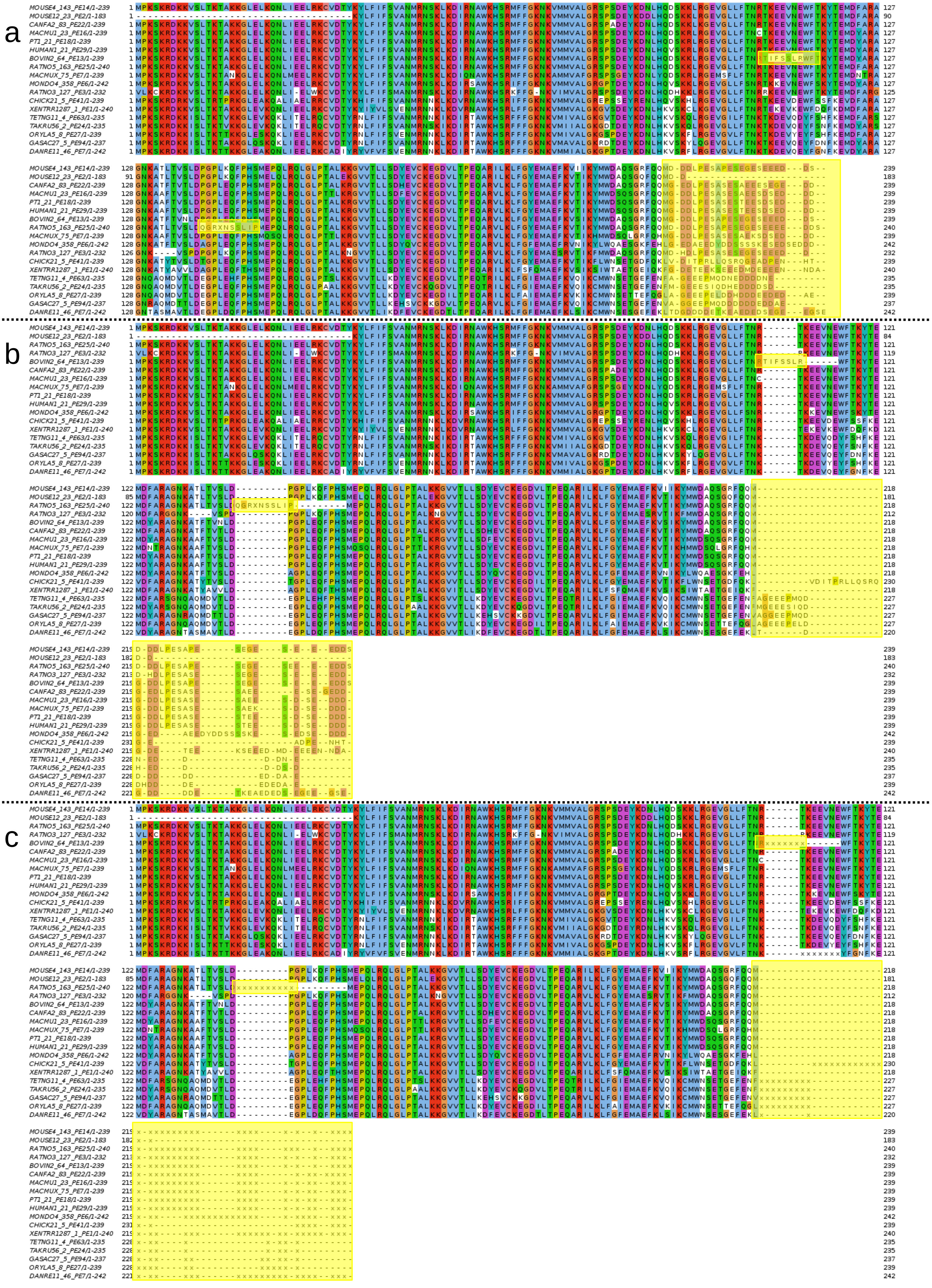

Figure S8. MSA filtering example. a) Before alignment with PAGAN. Problematic regions are highlighted in yellow (2 non-homologous regions and one region with repeats). b) After alignment with PAGAN. Non-homologous regions are almost entirely isolated from the rest. c) End of the pipeline. Non-homologous or difficult to align regions are masked with 'x', including the region containing repeats.
