# Supplementary material for: Detection of Pathways Affected by Positive Selection in Primate Lineages Ancestral to Humans
Source: Mol Biol Evol. 2017 Feb 25;34(6):1391–402. doi: 10.1093/molbev/msx083 (PMC5435107; doi:10.1093/molbev/msx083)
Supplement: Supplementary Data [file msx083_Supp.zip › Supplemental Material_MBE_Daub.pdf]

Detection of pathways affected by positive selection in primate lineages ancestral to humans

Daub, J.T., Moretti, S., Davidov, I. I., Excoffier, L. Robinson-Rechavi, M.

Supporting Information

Supplemental Text

Text S1 .... p. 2

Supplemental Tables

Table S1 .... in separate file, description on p. 5
Table S2 .... p. 6
Table S3 .... in separate file, description on p. 7
Table S4 .... in separate file, description on p. 8

Supplemental Figures

Figure S1 .... p. 9
Figure S2 .... p. 10
Figure S3 .... p. 11
Figure S4 .... p. 12
Figure S5 .... p. 13
Figure S6 .... in separate file, description on p. 14
Figure S7 .... p. 15
Figure S8 .... in separate file, description on p. 17
Figure S9 .... p. 18
Figure S10 .... p. 19
Figure S11 .... p. 20

Text S1

Using a parametric null distribution

If we assume independence between genes in a set, we can define the gene set level likelihoods, $\mathrm{LH0}_s$ and $\mathrm{LH1}_s$, as the product of the gene level likelihoods:

$$\mathrm{LH0}_s = \prod_{g \in set} \mathrm{LH0}_g, \quad \mathrm{LH1}_s = \prod_{g \in set} \mathrm{LH1}_g$$

We can then calculate a gene set level likelihood ratio test score ($\Delta \mathrm{lnL}_s$), which is equal to the sum of $\Delta \mathrm{lnL}_g$ scores of genes in the gene set:

$$\Delta \ln \mathrm{L}_s = 2\left(\sum_{g \in s} \ln \mathrm{LH1}_g - \sum_{g \in s} \ln \mathrm{LH0}_g \right) = \sum_{g \in s} \Delta \ln \mathrm{L}_g$$

This $\Delta \mathrm{lnL}_s$ score can then be compared against a parametric null distribution to infer its significance.

In the branch-site test for positive selection, the dN/dS ratio for the class of sites under positive selection (ω_2) is constrained by $\omega_2 \geq 1$ and the null model H_0 is nested in H_1 with ω_2 fixed on the boundary of the parameter space ($\omega_2 = 1$). Therefore, in case of no positive selection (H_0 is true), we would expect that the ΔlnL values at the gene level ($\Delta \mathrm{lnL}_g$) are distributed as a mixture of 50% zeros and 50% (Self and Liang 1987; Zhang et al. 2005). For the gene set level ΔlnL values ($\Delta \mathrm{lnL}_s$), we have *N* free parameters for a gene set of size *N*. The expected null distribution of $\Delta \mathrm{lnL}_s$ would be a mixture of χ^2 distributions with 0, 1, ... , *N* degrees of freedom, where the mixture weights are the binomial coefficients (Ota et al. 2000). For example, with gene sets containing three genes, the distribution would be:

$$\tfrac{1}{8}\chi_0^2 + \tfrac{3}{8}\chi_1^2 + \tfrac{3}{8}\chi_2^2 + \tfrac{1}{8}\chi_3^2$$

The probability for observing a $\Delta\ln L_s$ of x or higher in gene sets of size three can then be calculated as:

$$\Pr(\Delta \ln L_s > x) = \tfrac{1}{8}\Pr(\chi_0^2 > x) + \tfrac{3}{8}\Pr(\chi_1^2 > x) + \tfrac{3}{8}\Pr(\chi_2^2 > x) + \tfrac{1}{8}\Pr(\chi_3^2 > x)$$

The ΔlnLs scores that we use have a higher proportion of zero's, namely around 70-80%. Testing our gene sets against a parametric null distribution, results therefore in unacceptable p-value distributions, which is shown by the following qq-plots comparing the p-values from a parametric test with an expected uniform distribution:

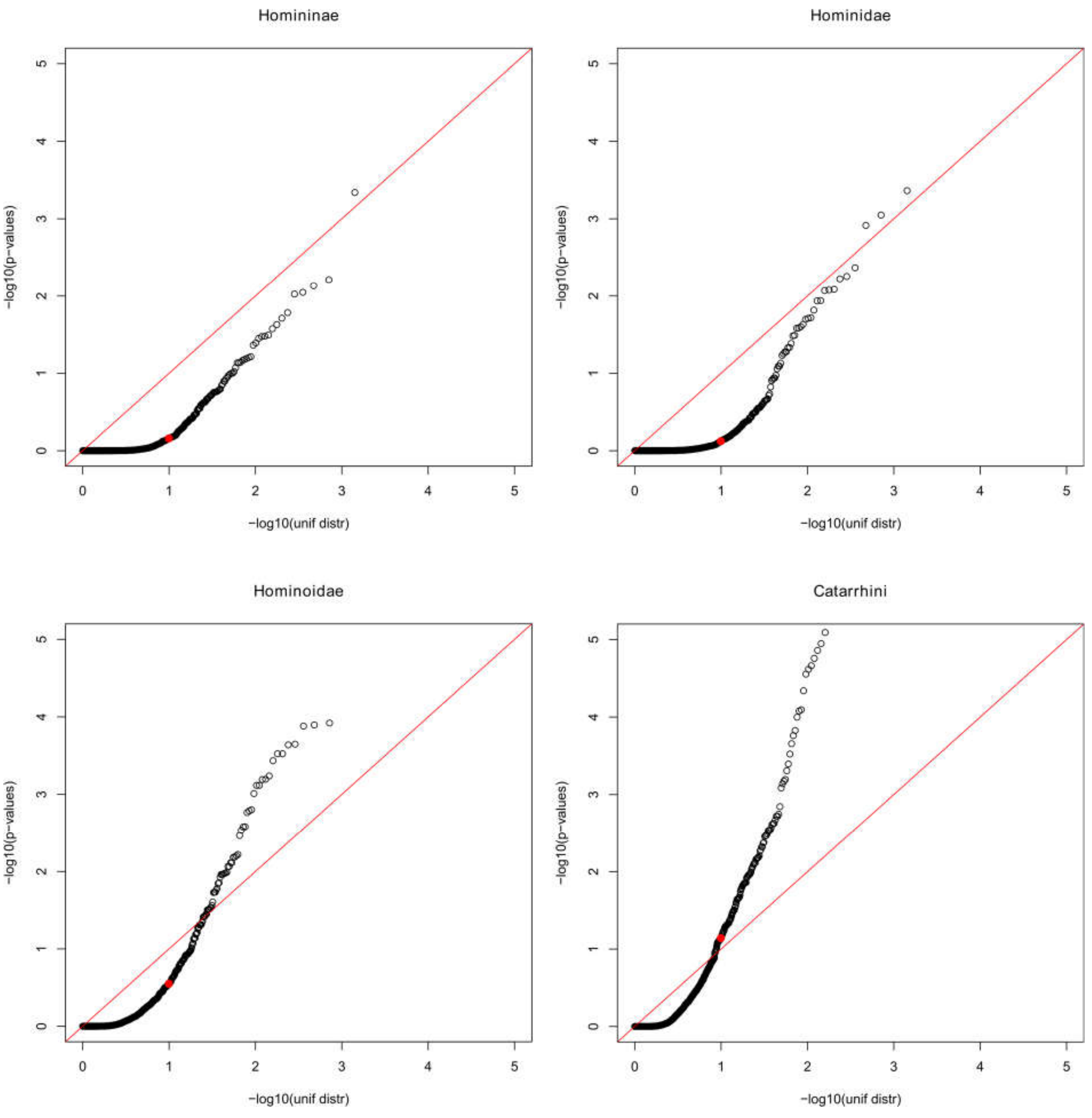
Homininae
Hominidae
Hominoidae
Catarrhini
−log10(p−values)
−log10(unif distr)
0
1
2
3
4
5

Table S1

This table is a separate excel file (TableS1.xlsx) which contains three tables: (A) Top scoring gene sets in the gene set enrichment test before pruning, their rank and score and p- and q-value (also after leaving out the highest scoring gene). All gene sets with $q<0.2$ are reported. (B) All significantly scoring pathways and their rank (before pruning) in all four branches. An '-' indicates that the set was not defined in that particular branch. Gene sets that scored significant after pruning are marked in bold. (C) Genes in top scoring gene sets and their ΔlnL4 and ΔlnL scores.

Table S2

A. Correlation (Pearson's r) between selection score ΔlnL4 and GC statistics at the gene level

**Per branch**	**r**	**[ci1, ci2]**	**p-value**
GC content	-0.045	[-0.054, -0.036]	<2.2e-16
**Per tree**	**r**	**[ci1, ci2]**	**p-value**
mean GC content	-0.043	[-0.052, -0.034]	<2.2e-16
GC variance	0.044	[0.036, 0.053]	<2.2e-16
max GC – min GC	0.074	[0.065, 0.083]	<2.2e-16

[ci1, ci2]: 95% confidence interval

B. Correlation (Pearson's r) between gene set enrichment score (-log[p-value]) and GC statistics at the gene set level. Significant correlations with |r| > 0.1 are marked in bold.

**Per branch**			
**GC content**	**r**	**[ci1, ci2]**	**p-value**
Homininae	-0.050	[-0.102, 0.002]	0.058
Hominidae	-0.068	[-0.121, -0.016]	0.011
Hominoidae	0.013	[-0.041, 0.066]	0.638
Catarrhini	-0.040	[-0.094, 0.014]	0.144
**Per tree**			
**mean GC content**	**r**	**[ci1, ci2]**	**p-value**
Homininae	-0.034	[-0.086, 0.018]	0.202
Hominidae	-0.066	[-0.118, -0.014]	0.013
Hominoidae	0.022	[-0.03, 0.074]	0.402
Catarrhini	-0.021	[-0.073, 0.03]	0.417
**GC variance**	**r**	**[ci1, ci2]**	**p-value**
Homininae	**0.183**	[0.132, 0.233]	3.83e-12
Hominidae	**0.172**	[0.121, 0.222]	5.87e-11
Hominoidae	**0.154**	[0.103, 0.204]	4.46e-09
Catarrhini	**0.186**	[0.135, 0.235]	1.18e-12
**max GC – min GC**	**r**	**[ci1, ci2]**	**p-value**
Homininae	0.033	[-0.019, 0.085]	0.217
Hominidae	-0.007	[-0.059, 0.045]	0.803
Hominoidae	-0.063	[-0.114, -0.011]	0.017
Catarrhini	0.018	[-0.034, 0.07]	0.493

[ci1, ci2]: 95% confidence interval

Table S3

This table is part of a separate excel file (TableS3_S4.xlsx) comparing results of the gene set enrichment test on a dataset of gene trees with or without duplications. It contains five tables: The number of available genes and gene sets for each branch excluding or including duplications (A), and -for each of the four tested branches- top scoring gene sets in the enrichment test with duplications and their score in the test without duplications (B-E).

Table S4

This table is part of a separate excel file (TableS3_S4.xlsx) presenting results of the gene set enrichment test where missing branches were not given the ΔlnL4 score of their child branch. It contains three tables: The number of available genes and gene sets for each branch excluding or including missing branches (using the ΔlnL4 score of their child branch) (A), and two tables with top scoring gene sets in the enrichment test before (B) and after (C) pruning, their rank and score and p- and q-value. All gene sets with q-value<0.2 are reported.

Figure S1

Proportion (A) and distribution (B) of non-zero ΔlnL4 scores in the genes in the four tested branches, split between genes that are not part of a gene set and genes that are part of at least one gene set. Especially the Homininae and Hominidae branches show a lower proportion and lower values of non-zero scores for genes in sets. The significance of the difference in distributions of non-zero ΔlnL4 scores is tested with a Wilcoxon signed-rank test.

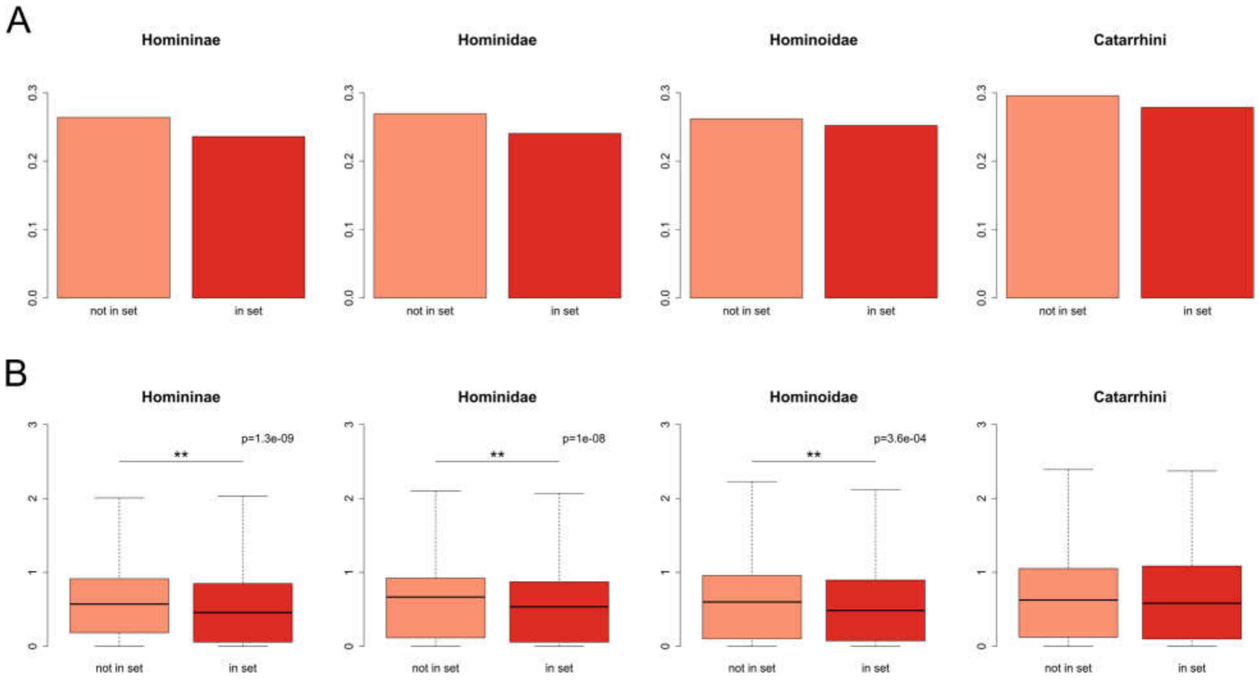

Figure S2

Enrichment map of the gene sets enriched for signals of positive selection in the Homininae branch. The nodes represent the 6 pathways with q-values < 0.2 before pruning (removal of overlapping genes). The node color scale represents gene set p-values. Edges represent mutual overlap; nodes are connected if one of the sets has at least 33% of its genes in common with the other gene set. The widths of the edges scale with the similarity between nodes. The two pathways that remained significant after pruning are marked with a black circle. A full-size version of this figure is available at https://dx.doi.org/10.6084/m9.figshare.3119029.

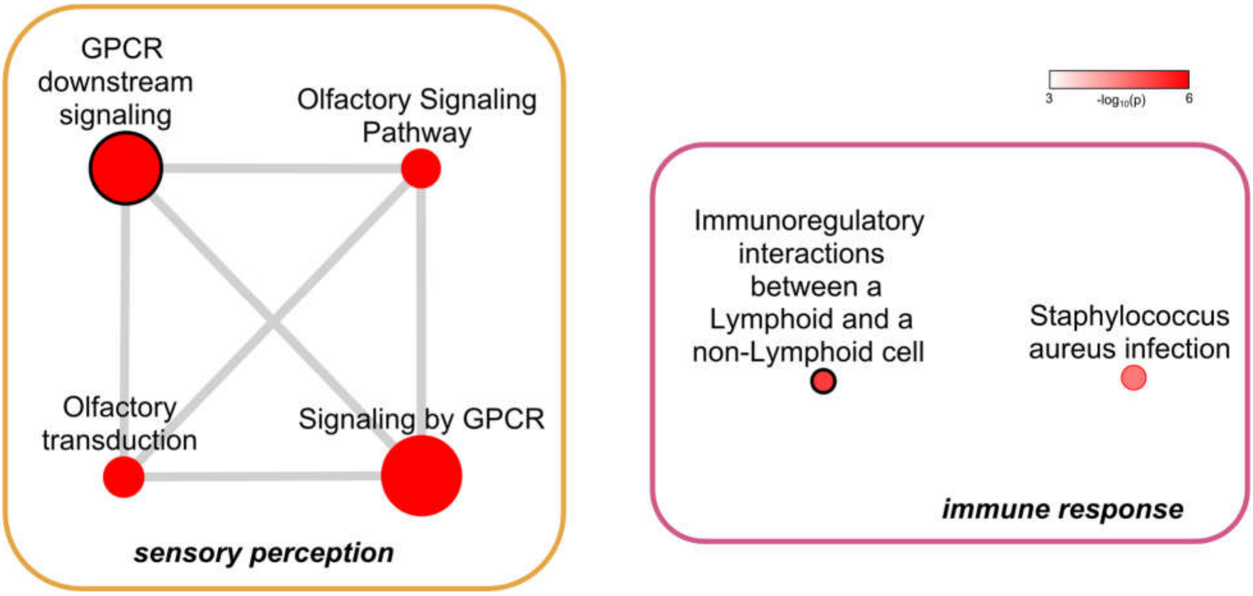

Figure S3

Enrichment map of the gene sets enriched for signals of positive selection in the Hominidae branch. The nodes represent the 32 pathways with q-values < 0.2 before pruning (removal of overlapping genes). The seven pathways that remained significant after pruning are marked with a black circle. See Figure S2 for a more detailed explanation of the enrichment map. Nodes marked with * represent unions of pathways that share more than 95% of their genes. A full-size version of this figure is available at https://dx.doi.org/10.6084/m9.figshare.3119029.

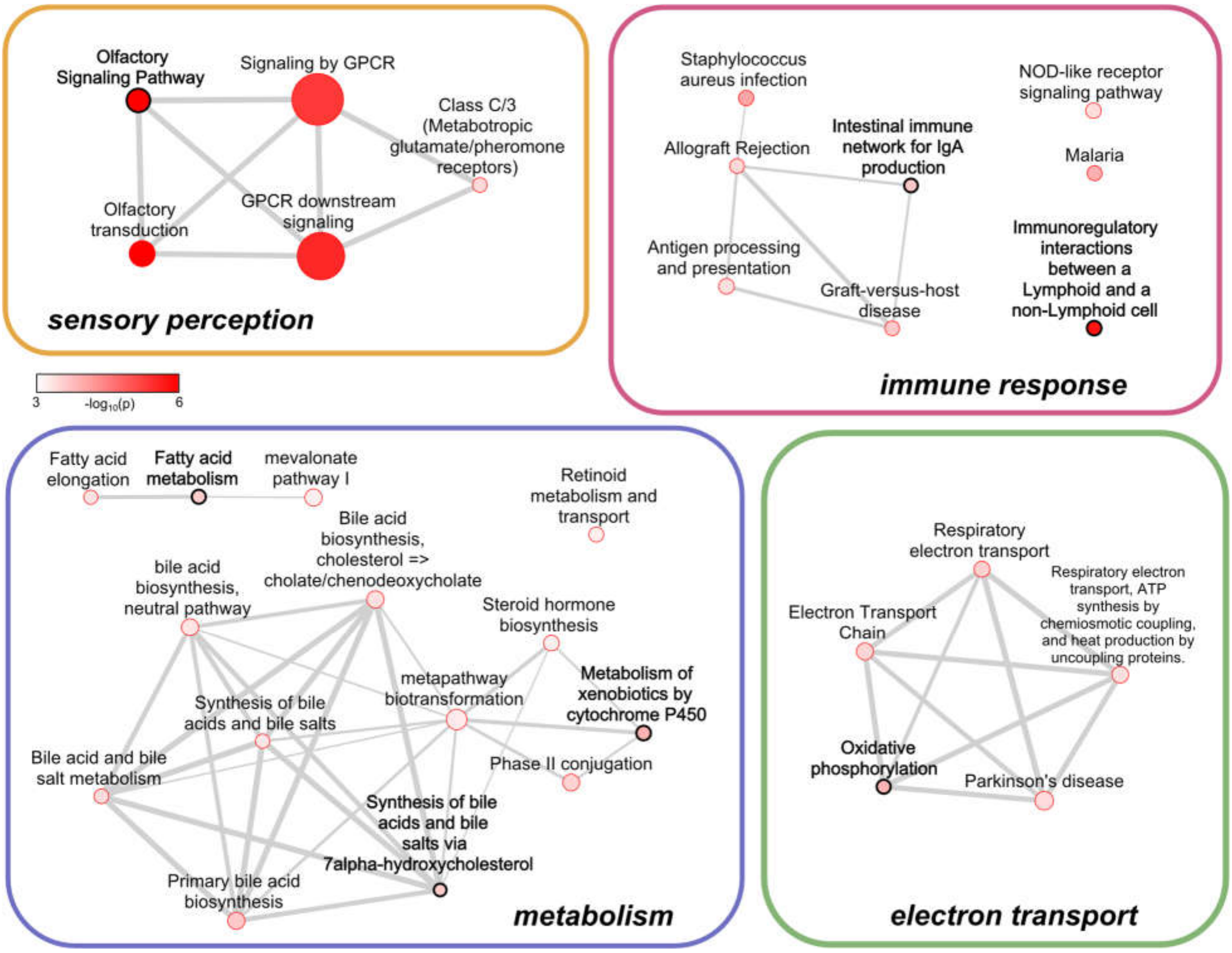

Figure S4

Enrichment map of the gene sets enriched for signals of positive selection in the Hominoidae branch. The nodes represent the 42 pathways with q-values < 0.2 before pruning (removal of overlapping genes). The six pathways that remained significant after pruning are marked with a black circle. See Figure S2 for a more detailed explanation of the enrichment map. A full-size version of this figure is available at https://dx.doi.org/10.6084/m9.figshare.3119029.

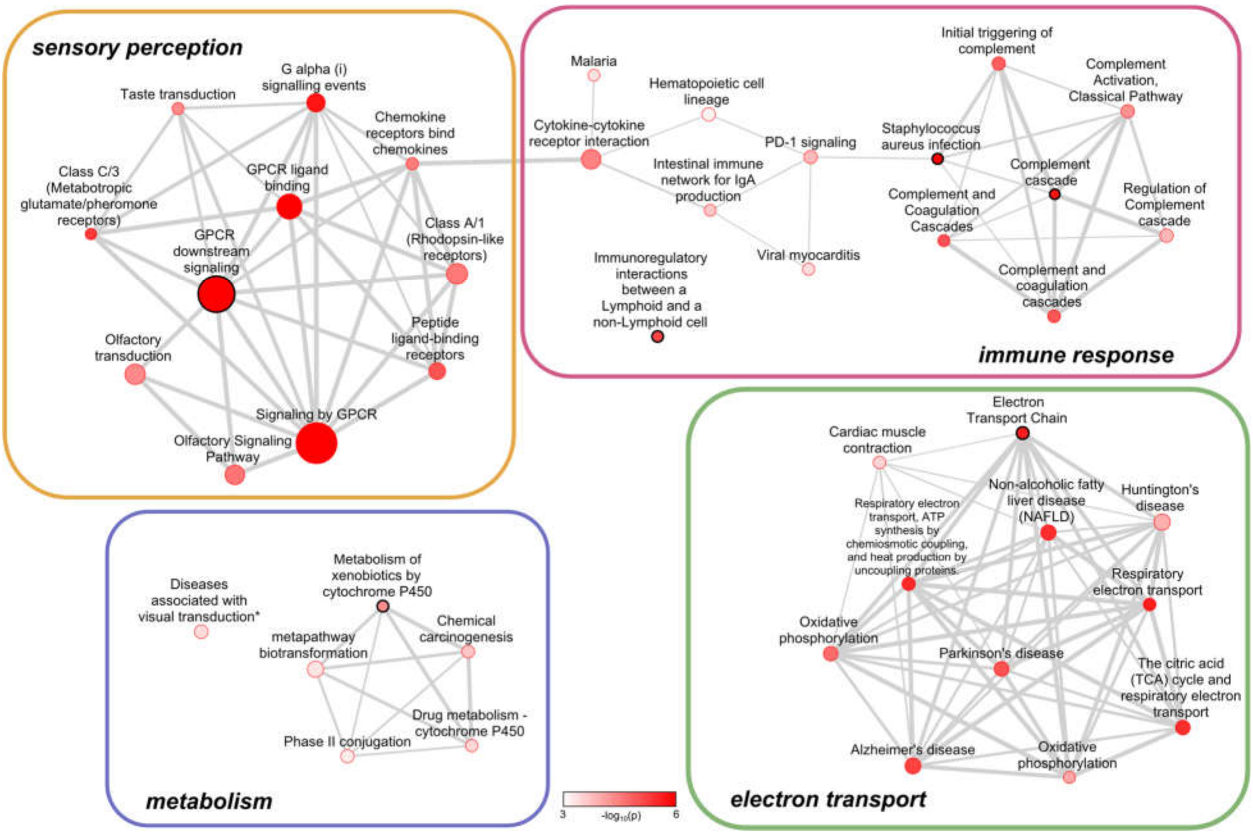

Figure S5

Enrichment map of the gene sets enriched for signals of positive selection in the Catarrhini branch. The nodes represent the 93 pathways with q-values < 0.2 before pruning (removal of overlapping genes). The nine pathways that remained significant after pruning are marked with a black circle. See Figure S2 for a more detailed explanation of the enrichment map. Nodes marked with * represent unions of pathways that share more than 95% of their genes. A full-size version of this figure is available at https://dx.doi.org/10.6084/m9.figshare.3119029.

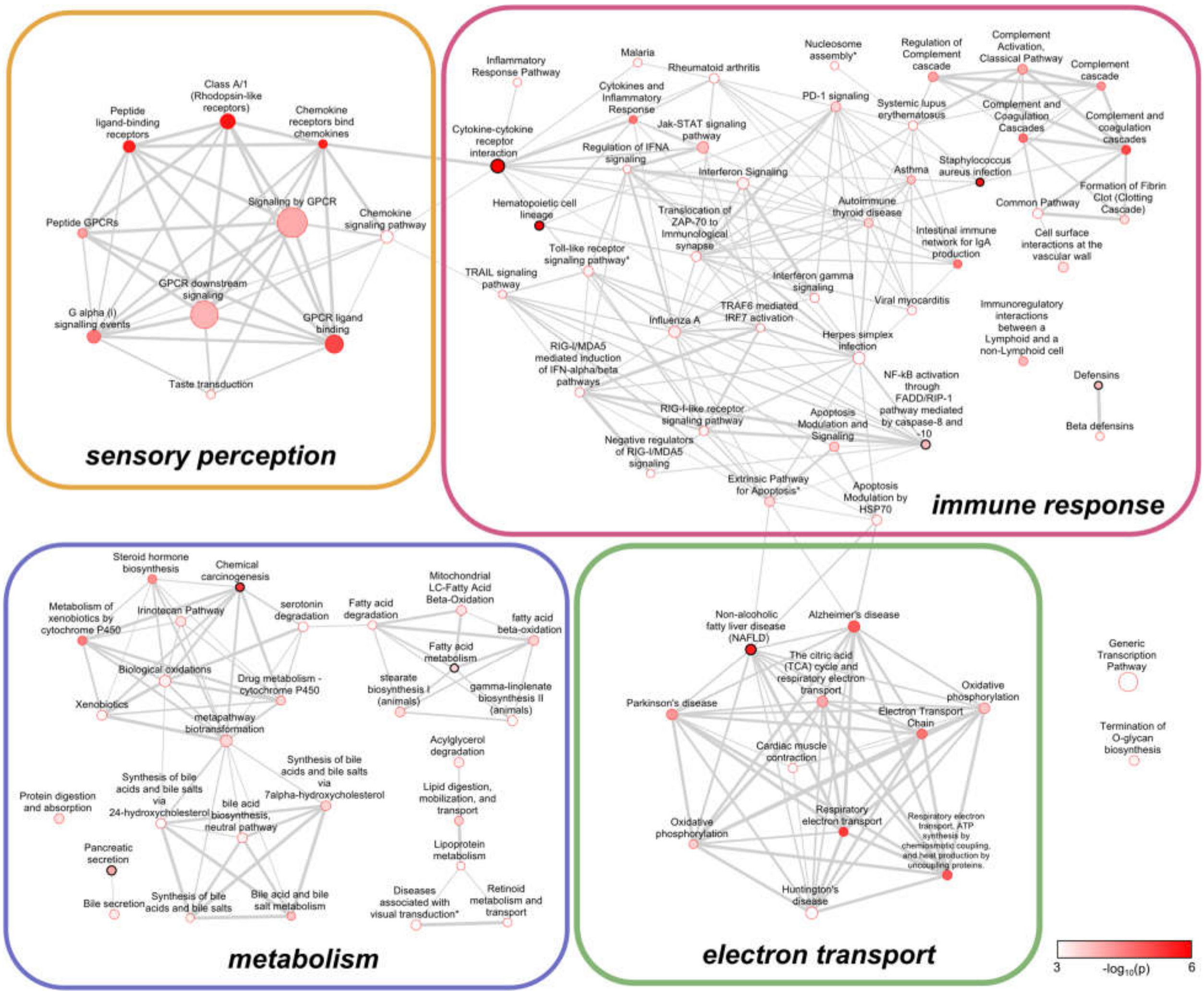

Figure S6

This figure is a separate pdf file (Figure S6.pdf) containing heat maps showing branch specific ΔlnL4 scores of genes in pathways that score significant ($q < 0.2$) in the gene set enrichment test after pruning. The ΔlnL4 score is computed as the fourth root of the log-likelihood ratio in the branch-site test for positive selection. Branches where a pathway scores significant are marked with a '*'. The genes are grouped by hierarchical clustering to visualize blocks with similar signals among branches. Genes for which ΔlnL4 scores were not available (NA) in a certain branch are depicted in grey. Genes are merged (horizontally) with their paralog(s) into an 'ancestral gene' in the branches preceding a duplication and their scores were included only once in the calculation of the SUMSTAT score for these branches. Genes with (vertically) merged branches represent cases where the sequence of one or more species is missing or excluded, resulting in a single 'average' ΔlnL4 score over multiple branches. We used this score when testing each branch separately. Full-size versions of these heat maps are available at https://dx.doi.org/10.6084/m9.figshare.3119026.

Figure S7

Enrichment map of gene sets enriched with genes that were excluded in Selectome. The nodes represent the 62 pathways with q-values < 0.2 in a Fisher's exact test. See Figure S2 for a more detailed description of the enrichment map. Nodes marked with * represent unions of pathways that share more than 95% of their genes. A full-size version of this figure is available at https://dx.doi.org/10.6084/m9.figshare.3119122.

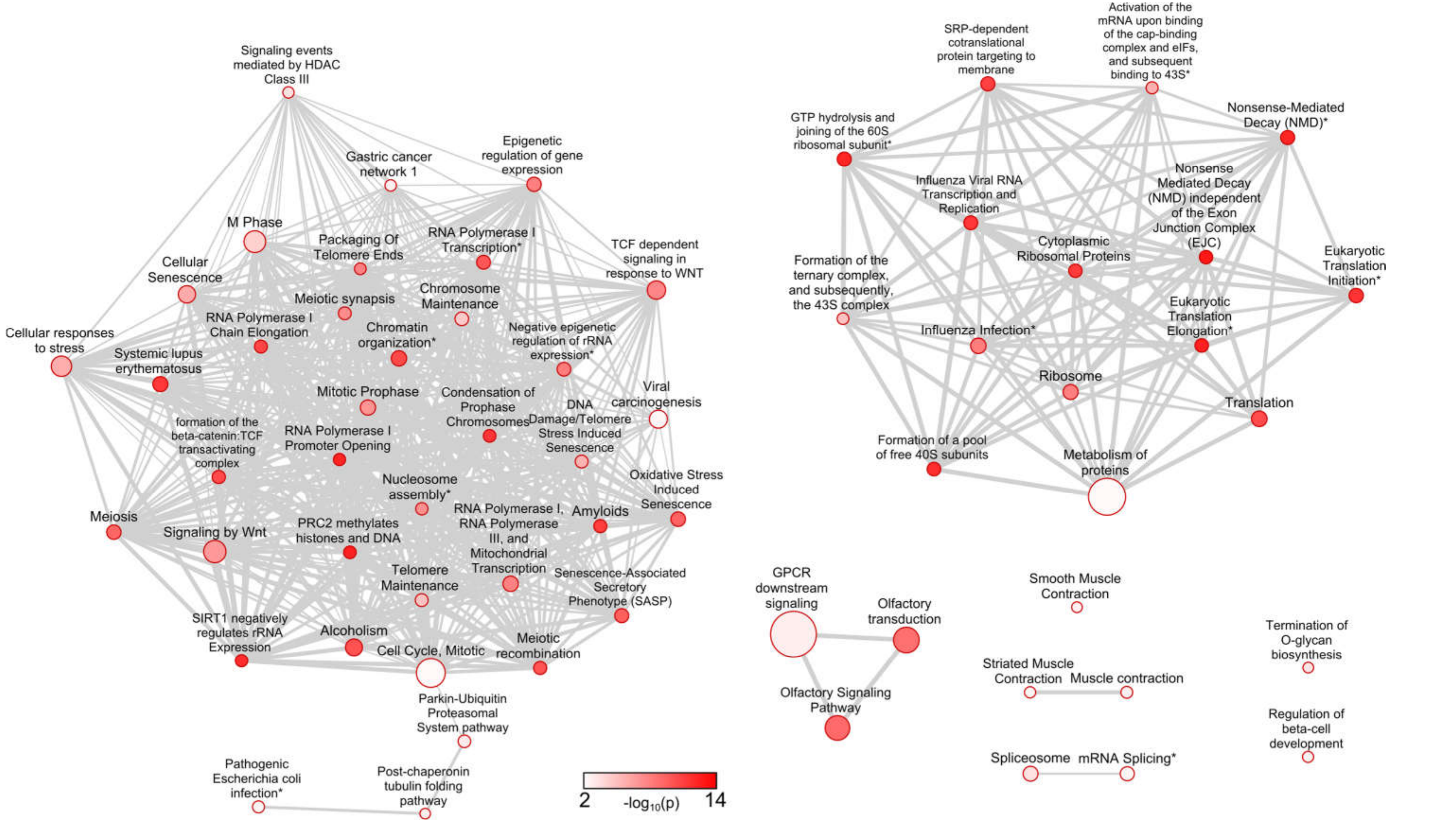

Signaling events mediated by HDAC Class III
Gastric cancer network 1
Epigenetic regulation of gene expression
M Phase
Packaging Of Telomere Ends
RNA Polymerase I Transcription*
TCF dependent signaling in response to WNT
Cellular Senescence
Meiotic synapsis
Chromosome Maintenance
RNA Polymerase I Chain Elongation
Chromatin organization*
Negative epigenetic regulation of rRNA expression*
Cellular responses to stress
Systemic lupus erythematosus
Mitotic Prophase
Condensation of Prophase Chromosomes
DNA Damage/Telomere Stress Induced Senescence
Viral carcinogenesis
formation of the beta-catenin:TCF transactivating complex
RNA Polymerase I Promoter Opening
Nucleosome assembly*
Oxidative Stress Induced Senescence
Meiosis
Signaling by Wnt
PRC2 methylates histones and DNA
RNA Polymerase I, RNA Polymerase III, and Mitochondrial Transcription
Amyloids
Telomere Maintenance
Senescence-Associated Secretory Phenotype (SASP)
SIRT1 negatively regulates rRNA Expression
Alcoholism
Cell Cycle, Mitotic
Meiotic recombination
Parkin-Ubiquitin Proteasomal System pathway
Pathogenic Escherichia coli infection*
Post-chaperonin tubulin folding pathway
2
-log10(p)
14
SRP-dependent cotranslational protein targeting to membrane
Activation of the mRNA upon binding of the cap-binding complex and eIFs, and subsequent binding to 43S*
Nonsense-Mediated Decay (NMD)*
GTP hydrolysis and joining of the 60S ribosomal subunit*
Influenza Viral RNA Transcription and Replication
Nonsense Mediated Decay (NMD) independent of the Exon Junction Complex (EJC)
Cytoplasmic Ribosomal Proteins
Formation of the ternary complex, and subsequently, the 43S complex
Eukaryotic Translation Initiation*
Influenza Infection*
Eukaryotic Translation Elongation*
Ribosome
Translation
Formation of a pool of free 40S subunits
Metabolism of proteins
GPCR downstream signaling
Olfactory transduction
Olfactory Signaling Pathway
Smooth Muscle Contraction
Striated Muscle Contraction
Muscle contraction
Termination of O-glycan biosynthesis
Regulation of beta-cell development
Spliceosome
mRNA Splicing*

Figure S8

This figure is a separate pdf file (Figure S8.pdf) showing an MSA filtering example. a) Before alignment with PAGAN. Problematic regions are highlighted in yellow (2 non-homologous regions and one region with repeats). b) After alignment with PAGAN. Non-homologous regions are almost entirely isolated from the rest. c) End of the pipeline. Non-homologous or difficult to align regions are masked with 'x', including the region containing repeats.

Figure S9

Example of multiple Homininae branches (branch *1a* and *1b*, marked red) in the *DMXL1* gene tree. Only the oldest branch (*1b*) will be used in the enrichment test. Table A illustrates that initially the Homininae branches occur in double in the table, while after removing the youngest Homininae branch, only one row per branch (taxon) and human gene remains (Table B). It should be emphasized that this is not a biological phenomenon, but simply a technical result of the labeling choices made in the Ensembl database, which are carried into the Selectome database and thus into the dataset used in this study.

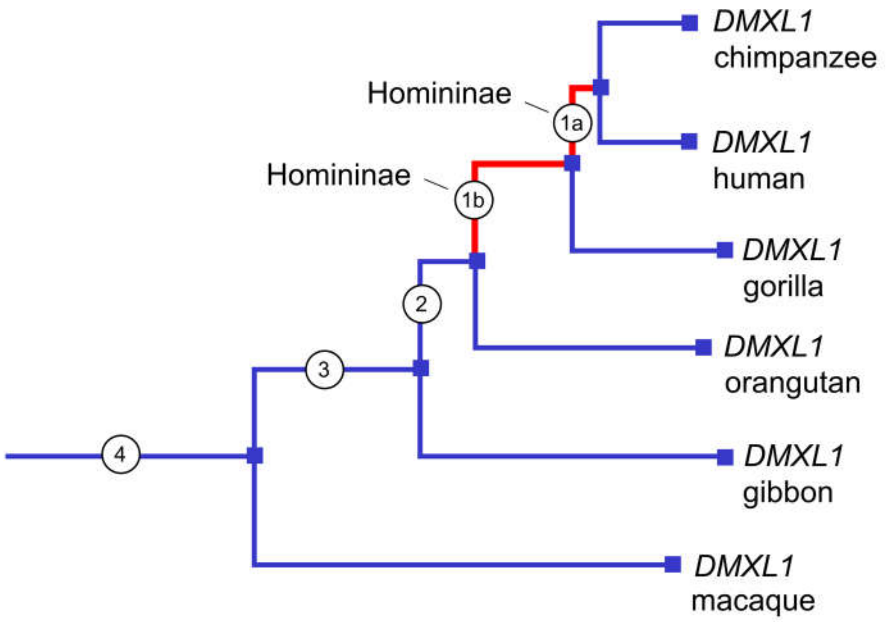

A

branch	taxon	gene	*score*
1a	Homininae	*DMXL1*	*0.00*
1b	Homininae	*DMXL1*	*0.00*
2	Hominidae	*DMXL1*	*0.00*
3	Hominoidae	*DMXL1*	*0.00*
4	Catarrhini	*DMXL1*	*0.80*

remove youngest branch

B

branch	taxon	gene	*score*
1b	Homininae	*DMXL1*	*0.00*
2	Hominidae	*DMXL1*	*0.00*
3	Hominoidae	*DMXL1*	*0.00*
4	Catarrhini	*DMXL1*	*0.80*

Figure S10

Example of a missing branch (Catarrhini) in the CE3 gene tree, due to an absent or low quality macaque sequence for this gene. The score assigned to the Hominoidae branch actually represents an 'average' score for both the Hominoidae and Catarrhini branch, and therefore we use this score for both branches in the enrichment test. Table A illustrates that initially one branch is missing, while after copying the Hominoidae score into a new Catarrhini row, one row per branch (taxon) and human gene is now available (Table B).

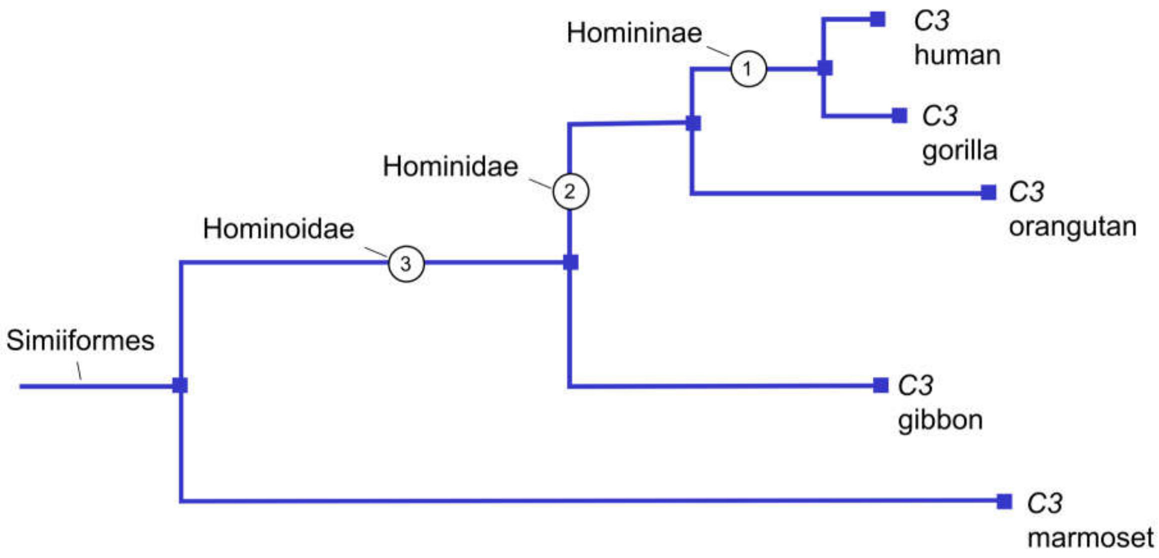

A

branch	taxon	gene	score
1	Homininae	C3	0.00
2	Hominidae	C3	0.62
3	Hominoidae	C3	1.35

copy into new branch

B

branch	taxon	gene	score
1	Homininae	C3	0.00
2	Hominidae	C3	0.62
3	Hominoidae	C3	1.35
4	Catarrhini	C3	1.35

Figure S11

Example of a duplication event (red node) in the Hominidae branch of the *CYP4A11*/*CYP4A22* gene tree. In the enrichment tests (i) the branch leading to the duplication (*2c*) is removed and (ii) in the Hominoidae (*3*) and Catarrhini branch (*4*), the 'ancestral' gene *CYP4A11*, *CYP4A22* replaces the two human paralogs *CYP4A11* and *CYP4A22*. Table A illustrates that initially some branches (and their scores) occur in double in the table. After merging and removing branches, only one row per branch (taxon) and human gene remains (Table B).

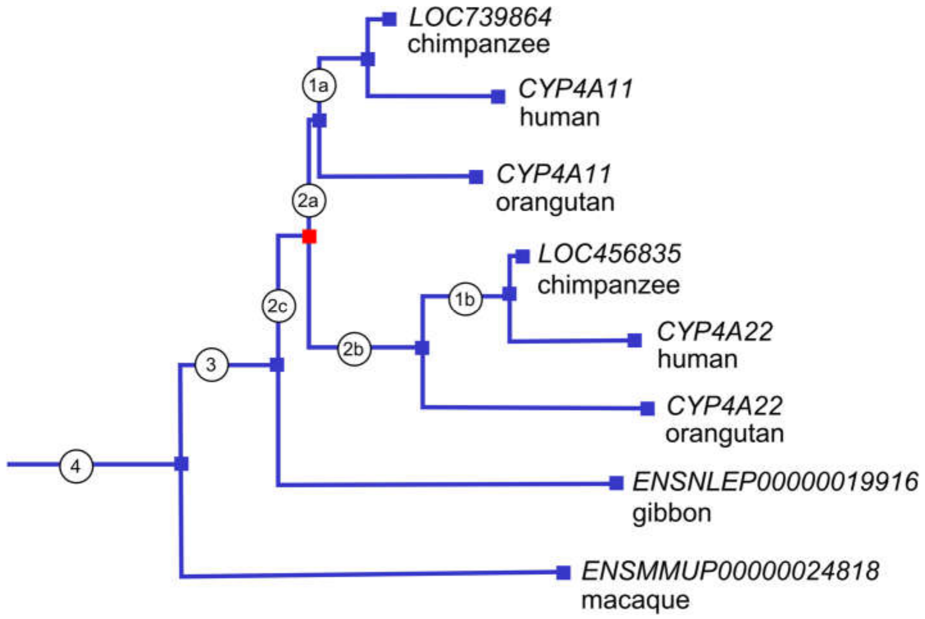

A

branch	taxon	gene	score
1a	Homininae	*CYP4A11*	*0.00*
1b	Homininae	*CYP4A22*	*0.00*
2a	Hominidae	*CYP4A11*	*0.00*
2b	Hominidae	*CYP4A22*	*0.57*
2c	Hominidae	*CYP4A11*	*0.00*
2c	Hominidae	*CYP4A22*	*0.00*
3	Hominoidae	*CYP4A11*	*0.00*
3	Hominoidae	*CYP4A22*	*0.00*
4	Catarrhini	*CYP4A11*	*0.00*
4	Catarrhini	*CYP4A22*	*0.00*

remove branch to duplication

merge with CYP4A11 into ancestral gene

B

branch	taxon	gene	score
1a	Homininae	*CYP4A11*	*0.00*
1b	Homininae	*CYP4A22*	*0.00*
2a	Hominidae	*CYP4A11*	*0.00*
2b	Hominidae	*CYP4A22*	*0.57*
3	Hominoidae	*CYP4A11, CYP4A22*	*0.00*
4	Catarrhini	*CYP4A11, CYP4A22*	*0.00*

References

Ota R, Waddell PJ, Hasegawa M, Shimodaira H, Kishino H. 2000. Appropriate likelihood ratio tests and marginal distributions for evolutionary tree models with constraints on parameters. Mol Biol Evol 17:798-803.

Self SG, Liang KY. 1987. Asymptotic Properties of Maximum-Likelihood Estimators and Likelihood Ratio Tests under Nonstandard Conditions. Journal of the American Statistical Association 82:605-610.

Zhang J, Nielsen R, Yang Z. 2005. Evaluation of an improved branch-site likelihood method for detecting positive selection at the molecular level. Mol Biol Evol 22:2472-2479.
